# Supplementary material for: Integrating Metabolomics and Gene Expression Underlying Potential Biomarkers Compounds Associated with Antioxidant Activity in Southern Grape Seeds
Source: Metabolites. 2023 Jan 31;13(2):210. doi: 10.3390/metabo13020210 (PMC9963462; doi:10.3390/metabo13020210)
Supplement: Supplementary file 1 [file metabolites-13-00210-s001.zip › Table S1.pdf]

Table S1. Identified metabolites in C5, C6, and LF muscadine ripe berries.

NO	Compounds identified in ESI+ mode	Compounds identified in ESI- mode	Compounds identified in ESI- &ESI+ mode
**1**	Trehalose 6-phosphate	16-Hydroxyhexadecanoic acid	12-Hydroxydodecanoic acid
**2**	2,4,6-Octatriynoic acid	3,5-Digalloylepicatechin	2-Furoic acid
**3**	2,5-Furandicarboxylic acid	3-Oxohexadecanoic acid	2-Methylbenzoic acid
**4**	2-Deoxymugineic acid	5-Methoxysalicylic acid	4-Acetamidobutanoic acid
**5**	2-Furanmethanol	6-Chlorocatechin	4-Dodecylbenzenesulfonic Acid
**6**	2-Isopropyl citrate	6-Epi-7-isocucurbic acid glucoside	4-Hydroxybenzaldehyde
**7**	2-Methylaconitate	7-Methylxanthine	4-Pyridoxic acid
**8**	2-Methylcitric acid	Aesculin	Abscisic acid
**9**	3 Hydroxycoumarin	Ascorbic acid	Aconitic acid
**10**	3,4-Dihydroxymandelic acid	Aucubin	Adipic acid
**11**	3-O-p-Coumaroylquinic acid	Azelaic acid	Arbutin
**12**	3-Oxododecanoic acid	beta-D-Glucose 6-phosphate	Asiatic acid
**13**	3-trans-p-Coumaroylrotundic acid	Caryoptosidic acid	Astilbin
**14**	4-Guanidinobutanoic acid	Cibaric acid	Caffeic acid
**15**	4-Hydroxynonenal	Citramalic acid	Caffeoylmalic acid
**16**	5-Aminolevulinic acid	Citrinin	Catechin
**17**	5-Hydroxy-2-furoic acid	Cyclodopa glucoside	Chlorogenic acid
**18**	5-Hydroxyindoleacetic acid	Delphinidin 3-lathyroside 5-glucoside	Cinnamic acid
**19**	8,8-Methylenebiscatechin	Delphinidin 3-sophoroside	Citric acid
**20**	9-Oxo-nonanoic acid	Deoxyribose	Cysteine
**21**	Acetophenone	D-Glucuronic acid	Delphinidin 3-arabinoside
**22**	Acetylornithine	Diflunisal	Deoxyinosine
**23**	Adenosine	Epiafzelechin 3-gallate	Dicaffeoylquinic acid
**24**	Alanine	Epigallocatechin 3,5-di-gallate	Ellagic acid
**25**	Amidosulfonic acid	Epigallocatechin gallate	Epicatechin-3-gallate
**26**	Aminobutyric acid	Eujambolin	Epigallocatechin
**27**	Arecoline	Galactaric acid	Epigallocatechin 3-cinnamate
**28**	Argininosuccinic acid	Gentisic acid	Epigallocatechin 3-glucuronide
**29**	Asparagine	Gluconic acid	Fertaric acid
**30**	Benzaldehyde	Glyceric acid	Ferulic acid
**31**	Benzophenone	Hesperidin	Fumaric acid
**32**	Caftaric acid	Hydroxypropionic acid	Galactose
**33**	Capryloylglycine	Isolimonic acid	Gallic acid
**34**	Carvone	Isowertin 2-rhamnoside	Glucobrassicin
**35**	Citral	Kaempferol 3-xylosylglucoside	Glutamine
**36**	Coumaperine	Kaempferol 7-(6-galloylglucoside)	Glutathione
**37**	Coumarin	L-Cystine	Glycyrrhetinic acid
**38**	Cyclamic acid	Liqcoumarin	Guanosine
**39**	Cyclohexylamine	Lucuminic acid	Isochesnatin
**40**	Cytidine	Luteolin	Isocrotonic acid
**41**	Daucic acid	Malonic acid	Isoferulic acid 3-O-glucuronide
**42**	Diaminopimelic acid	Methyl acetate	Isolariciresinol 4-O-beta-D-glucoside
**43**	Dihydroferulic acid	Methylsuccinic acid	Isorhamnetin 3-(6-malonylglucoside)
**44**	Dihydrojasmonic acid	Naringin	Itaconic acid
**45**	Dihydromelilotoside	Neuraminic acid	Jasmonic acid
**46**	Dihydrosinapic acid	N-Undecylbenzenesulfonic acid	Kaempferol 3-glucuronide
**47**	Dihydrotestosterone	Oxalacetic acid	L-Arginine
**48**	Dihydrothymine	Oxalic acid	L-Aspartic acid
**49**	Dihydrouracil	Phenylacetaldehyde	Lepidimoic acid
**50**	Dopa	p-Hydroxyphenylacetic acid	L-Glutamic acid
**51**	Dopamine glucuronide	Piceid	L-Histidine
**52**	Eicosadienoic acid	Pimelic acid	Limocitrin 3-rutinoside
**53**	Eicosapentaenoic acid	Pyruvic acid	Linamarin

**54**	Epidermin	Retinoic acid	Linoleic acid
**55**	Epsilon-caprolactam	Rhamnazin 3-rutinoside	L-Leucine
**56**	Erucic acid	S-Lactoylglutathione	L-Phenylalanine
**57**	Erythrose	Stearic acid	L-Quebrachitol
**58**	Ethyl lactate	Suberic acid	L-Threonine
**59**	Ethyl oleate	Sucralose	L-Tyrosine
**60**	Folinic acid	Tartaric acid	Luteone
**61**	Fukiic acid	Threonic acid	Malic acid
**62**	Furaneol	Troxerutin	Medicagenic acid
**63**	gamma-Glutamylleucine	Undecanedioic acid	Mevalonic acid
**64**	Glucogallin	Ureidopropionic acid	Monobutylphthalate
**65**	Glucosamine	Uridine 5-diphosphate	Myricetin
**66**	Glucose 1-phosphate	Vanillic acid 4-O-sulfate	Myricetin 3-galactoside
**67**	Glutaric acid	Xanthylic acid	Myrsinone
**68**	Glycerol 3-phosphate		N-Acetyl-L-glutamic acid
**69**	Glycerophosphocholine		Naringenin
**70**	Guaiacol		Neoglucobrassicin
**71**	Guanine		N-Undecanoylglycine
**72**	Heptanal		Oleanolic acid
**73**	Hexanal		Ornithine
**74**	Histamine		Oxoadipic acid
**75**	Homomethionine		Oxoglutaric acid
**76**	Indole		Pantothenic acid
**77**	Indole-3-carboxaldehyde		Phaseolic acid
**78**	Isobutyric acid		Phloretin
**79**	Isorhamnetin		Phosphate
**80**	Isovanillic acid		Phosphoenolpyruvic acid
**81**	Kaempferol		Phthalic acid
**82**	Kojic acid		Piscidic acid
**83**	Kynurenic acid		Procyanidin B1
**84**	Lactosamine		Procyanidin C1
**85**	L-Agaritine		Protohypericin
**86**	Levulinic acid		Punicic acid
**87**	Linoleamide		Pyrocatechol
**88**	Linoleic acid		Pyroglutamic acid
**89**	Linoleoyl ethanolamide		Quercetin
**90**	L-Methionine		Quercetin 3-(6-malonyl-glucoside)
**91**	Lysine		Quercetin 3-arabinoside
**92**	Methionine sulfoxide		Quercetin 4-glucoside
**93**	Methyl cinnamate		Quinic acid
**94**	Muramic acid		Rutin
**95**	N-Acetylneuraminic acid		Salicylic acid
**96**	N-Acetylserotonin		Scopoletin
**97**	N-Acetylvaline		Serine
**98**	Neocnidilide		Shikimic acid
**99**	N-Glycolylneuraminic acid		Sinapic acid
**100**	Niacinamide		Succinic acid
**101**	Nicotinic acid		Syringic acid
**102**	N-Undecanoylglycine		Tocopheronic acid
**103**	Octadecanamide		Traumatic acid
**104**	Octadecylamine		Trehalose
**105**	Oleamide		Trifolin
**106**	Oleoylethanolamide		Tryptophan
**107**	Olopatadine		Uridine
**108**	Palmitic amide		
**109**	Palmitoleoylethanolamde		

**110**	Panthenol		
**111**	Pantoyllactone glucoside		
**112**	p-Coumaric acid		
**113**	p-Cresol		
**114**	p-Cymene		
**115**	Pelargonidin 3-rhamnoside		
**116**	Penmacric acid		
**117**	Phenol		
**118**	Phenylethylamine		
**119**	Phenylglyoxylic acid		
**120**	Phosphoglycolic acid		
**121**	Phosphohydroxypyruvic acid		
**122**	Phytosphingosine		
**123**	Pipecolic acid		
**124**	Piperidine		
**125**	Pirbuterol		
**126**	Proanthocyanidin A2		
**127**	Proline		
**128**	Prostaglandin B1		
**129**	protocatechuic acid		
**130**	Pubescenol		
**131**	Pyridoxal		
**132**	Pyruvic acid		
**133**	Quinolinic acid		
**134**	Quinone		
**135**	Resveratrol		
**136**	Rhamnitol		
**137**	Rutagravine		
**138**	Sebacic acid		
**139**	Spermidine		
**140**	Sphinganine		
**141**	Sphingosine		
**142**	Stearidonic acid		
**143**	Styrene		
**144**	Succinylacetone		
**145**	Targinine		
**146**	Taurine		
**147**	Theaflavic acid		
**148**	Thymine		
**149**	Tiglic acid		
**150**	Tridecanoic acid		
**151**	Triethylamine		
**152**	Tyrosine methylester		
**153**	Tyrosol		
**154**	Urocanic acid		
**155**	Valine		
**156**	Valylproline		
**157**	Vanillin		
