# Supplementary material for: Integrating Metabolomics and Gene Expression Underlying Potential Biomarkers Compounds Associated with Antioxidant Activity in Southern Grape Seeds
Source: Metabolites. 2023 Jan 31;13(2):210. doi: 10.3390/metabo13020210 (PMC9963462; doi:10.3390/metabo13020210)
Supplement: Supplementary file 1 [file metabolites-13-00210-s001.zip › Table S2.pdf]

Table S2. The annotation and oligonucleotide primers of assessed genes by qPCR.

Order	Gene ID	Gene Annotation	Gene Abbreviation
1	evm.model.chr14.2977	3-dehydroquinate synthase	DHQS
2	evm.model.chr14.559	3-dehydroquinate dehydratase	DHD
3	evm.model.chr18.2392	Shikimate kinase	SK
4	evm.model.chr14.3086	Chorismate mutase	CM
5	evm.model.chr6.362	Phenylalanine ammonia-lyase	PAL
6	evm.model.chr14.2273	Chalcone synthase	CHS
7	evm.model.chr13.1853	Cinnamoyl-CoA reductase	CCR
8	evm.model.chr4.2404	Cinnamyl alcohol dehydrogenase	CAD
9	evm.model.chr17.1013	Flavanone-3-hydroxylase	F3H
10	evm.model.chr18.1438	Dihydroflavonol 4-reductase	DFR
11	evm.model.chr17.567	Leucoanthocyanidin reductase	LAR
12	evm.model.chr10.496	Anthocyanidin reductase	ANR
13	evm.model.chr13.1779	gallate 1-β-glucosyltransferase	UGT
14	evm.model.chr16.1632	UDP-glucose: flavonoid 3-O-glucosyltransferase	UFGT
15	evm.model.chr18.2304	Laccase	LAC
16		Elongation Factor 1	EF1
17		Actin	Actin

Primer_F	Primer_R
CTTCTTCGGTTACATCGGCG	AAGTGTTCCATCCCCTCTCG
AGGAACTTCAGTCCTGCACA	GCCTTCATACTGACCACCCT
GACGAGTGTCTTTGGGGATT	TCCCATCATTCCAACAAGAA
GCAGGCTCTGTCAAAGAGAA	CCGTAGGCTTTGGTTTTCAT
CATTGGGAAGCTCATGTTTG	GGCTAGGAATTGGAGCTCAG
GTTTTGGTCGTCTGCTCTGA	TCTGAGCTGCAGAGACGAGT
CGTCTATGTCTGCGGTGGA	AACTTCCAAGCAGCCTCCT
GGAGAATTACTGCCCCAAAA	CTGTGATCCCAGCACAGAGT
CATATCCGACAGGAGGAGGT	TCCACCACCATCTCTTTGAA
TCGGTTCATGGCTGGTCAT	TGAGGCTTGGAGGCATTGA
AACCGTGGAAGAAGATGACC	ACTGCATCTCCGGATAAAGG
CAGAAGGGCTATGCTGTCAA	GACAAAGTCGCAACCTGCTA
ACCTCGACCCCATCAAAGG	TTCCGGGCCTTGAAGAAGT
TGAAGCGCTTGAAATACCAG	CGGATCAACCACATCTTCTG
ATCGTCCTTGCATGTTGGTA	TGACGATGCGGAGAAGATAG
CACTACTGCTGAACGGGAAA	GGACTTCTGGACAACGGAAT
CACTACTGCTGAACGGGAAA	GGACTTCTGGACAACGGAAT
